# Simultaneous emission of orthogonal handedness in circular polarization from a single luminophore

**DOI:** 10.1038/s41377-019-0232-0

**Published:** 2019-12-12

**Authors:** Kyungmin Baek, Dong-Myung Lee, Yu-Jin Lee, Hyunchul Choi, Jeongdae Seo, Inbyeong Kang, Chang-Jae Yu, Jae-Hoon Kim

**Affiliations:** 1 Department of Electronic Engineering, Hanyang University, 222 Wangsimni-ro, Seongdong-gu, Seoul 04763 Republic of Korea; 2 LG Display Co., Ltd., LG Science Park, 30 Magokjungang 10-ro, Gangseo-gu, Seoul 07796 Republic of Korea

**Keywords:** Polymers, Photonic devices

## Abstract

The direct emission of circularly polarized (CP) light improves the efficiency of an organic light-emitting diode and characterizes the secondary structure of proteins. In most cases, CP light is generated from a luminescent layer containing chiral characteristics, thereby generating only one kind of CP light in an entire device. Here, we propose direct CP light emissions using a twisted achiral conjugate polymer without any chiral dopant as an emitting layer (EML). The twisted structure is induced in the mesogenic conjugate polymer due to its elasticity by applying different alignment directions to its upper and lower interfaces. Furthermore, we demonstrate the simultaneous emission of orthogonal CP light in a single luminescent device by patterning different alignment directions on the surfaces of the EML. The light source with multipolarization including the orthogonal CP states is applicable to many applications in biosensors and optical devices.

## Simultaneous right and left circular polarization from single device

Both right- and left-handed circularly polarized light were generated from a single device, paving the way for novel applications in biosensors and organic LEDs. Chang-Jae Yu of Hanyang University in Korea and colleagues fabricated the device by using the liquid crystal phase of a polymer, called F8BT, which is commonly used in organic light-emitting diodes. They controlled the alignment of the molecules inside the polymer by rubbing its upper and lower surfaces in specific directions. The rubbing caused the molecules inside the polymer to align and twist. Light passing through the polymer simultaneously emerges as right- and left-handed circularly moving electromagnetic waves. The study demonstrates the feasibility of a light source with multiple polarizations, which could have applications in a variety of optical devices.

## Introduction

Control of the polarization of light is a key feature for displays, optical data storage, optical quantum information, and chirality sensing^1–4^. In many cases, polarized light is obtained through anisotropic absorption or selective reflection of unpolarized or partially polarized light^5–7^. The polarization can be controlled by propagation of the polarized light through birefringent materials such as liquid crystals (LCs)^8^. In other cases, polarized light is generated from uniaxially aligned luminophores using anisotropic alignment layers such as rubbed polyimide^9,10^. In particular, the direct emission of circularly polarized (CP) light has attracted great interest because of the enhanced performance of organic light-emitting diodes (OLEDs) and light sources for characterizing the secondary structure of proteins^11–13^. In a conventional OLED, since a circular polarizer in front of the OLED panel is inevitably required to prevent reflection of ambient light from a metal electrode, only half of the light extracted from the OLED panel reaches the eye. That is, the maximum efficiency of the emitted light is approximately 50%, even when other losses such as internal reflection are not taken into account. As a result, direct emission of CP light from an OLED with the same handedness as that of the circular polarizer in front of the OLED panel can increase the efficiency of the emitted light (see Fig. S1 in Supplementary Information). The degree of circular polarizations is defined by the dissymmetry factor, *g* = 2(*I*_L_ *−* *I*_R_)/(*I*_L_ + *I*_R_), where *I*_L_ and *I*_R_ denote the intensities of left-handed (LH) and right-handed (RH) CP light, respectively.

CP light can be generated by the intrinsic properties of used materials such as chiral luminophores^14–19^ or the extrinsic properties of macroscopic conformations such as the propagation of light through the twisted stacking of birefringent materials^20–22^. Regardless, to actually produce the CP light, the luminescent layer should contain chiral characteristics, which can be achieved, for example, by decorating the luminophores with chiral materials^16–18,22^ or doping chiral molecules into achiral materials^11,20,23,24^. To achieve a high dissymmetry factor, the twisted stacking of birefringent materials such as liquid crystals by doping chiral molecules has been deeply studied^11^. However, such chirality of the luminescent layer makes it possible to generate only one kind of CP light in an entire device since it is difficult to control the chiral sense spatially. In addition, the chirality of the dopant appears only in a limited temperature range. Although generating single CP light is important for improving the performance of OLEDs, the generation of orthogonal CP light in a single luminophore can pave the way towards novel light sources for biosensors^12,13^ and stereoscopic three-dimensional displays by using a single emitting layer without any additional optical film^25^. To produce various CP states including orthogonal CP light in a single device, a CP light-emitting device without any chiral part should be implemented.

Here, for the first time, we propose direct CP light emissions by using a twisted achiral conjugate polymer without any chiral dopant. The twisted structure is induced in an emissive layer (EML) by applying different boundary conditions to the upper and lower interfaces of the conjugate polymer with an LC phase. The different alignment directions on the upper and lower interfaces of the mesogenic EML promote a twisted configuration in the LC phase due to its elasticity^26^. Consequently, by applying a multialignment method onto surfaces of the EML, we demonstrate the simultaneous emission of orthogonal CP light in a single luminescent device. The linearly polarized (LP) light generated at the aligned mesogenic conjugate polymer is converted into different CP states by passing through the different twisted structures. For the leftward and rightward twisted domains in a single device, the dissymmetry factors (*g*_PL_) of the photoluminescence (PL) process are observed to be 0.60 and −0.63, respectively, showing opposite signs. Likewise, the dissymmetry factors (*g*_EL_) of the electroluminescence (EL) process are observed to be 0.57 and −0.64, respectively.

## Results

**Fig. 1:**
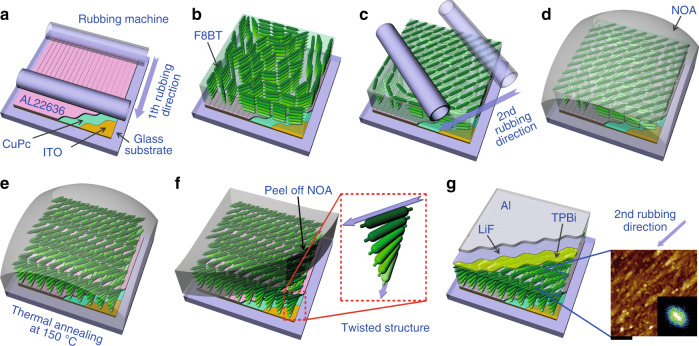
Schematic diagrams of the fabrication process. **a** The first rubbing of AL22636 coated on CuPc. **b** Spin coating and drying of F8BT layer, and **c** rubbing the F8BT (second rubbing) with different direction from the first rubbing. **d** Coating NOA on the rubbed F8BT and **e** thermal annealing the sample at mesomorphic temperature of the F8BT. **f** Cooling down the sample and peeling off NOA, and **g** TPBi/LiF/Al deposition in vacuum, sequentially. An AFM image and the corresponding Fourier-transformed image show the second rubbed surface of the F8BT. Here, scale bar represents 5 μm and arrows indicate the rubbing directions.

We used the achiral conjugate polymer poly(9,9-di-n-octylfluorenyl-2,7-diyl)-alt-(benzo[2,1,3]thiadiazol-4,8-diyl)] (F8BT) with an LC phase. To produce a twisted structure without any chirality, we introduced different rubbing directions to the upper and lower surfaces of the F8BT layer forming the EML, as shown in Fig. 1. After spin-coating F8BT on the rubbed polyimide (PI) (the first rubbing direction) film, determining the boundary condition of the lower surface (Fig. 1a), the boundary condition of the upper surface was established by rubbing the coated F8BT itself (the second rubbing direction) in a direction different from the PI rubbing (Fig. 1b, c). Here, the rubbing process on the PI produces a groove structure (surface anisotropy) and promotes the unidirectional alignment of LC materials on the rubbed PI layer^27,28^. In addition, based on a diagram of the corresponding energy levels, the PI acts as an electron blocking layer and a hole transfer layer as well as an alignment layer (see Fig. S2 in Supplementary Information). To achieve various twisting angles, we rubbed the F8BT layer at various angles with respect to the first rubbing direction. Thereafter, the upper surface was coated with UV epoxy (NOA65) as a protecting film to maintain the upper boundary condition up to the temperature at which the F8BT layer had an LC phase (Fig. 1d). The F8BT rubbing process also produces a groove structure on the upper F8BT surface. However, such surface grooves disappear after thermal annealing without any supporting film due to melting to the LC phase (see Fig. S3 in Supplementary Information). To retain the grooves on the upper F8BT surface, we introduced UV-curable resin onto the rubbed F8BT. The cured resin replicates the groove structure of the upper F8BT surface, and thus, the grooves on the upper F8BT surface are still maintained after thermal annealing. It should be noted that the direction of the grooves on the upper F8BT surface differs from that on the lower F8BT surface produced by the rubbed PI. Then, the sample was thermally annealed at 150 °C for 10 min to produce the twisted structure due to the elastic properties of the LC polymer (Fig. 1e). The continuously twisted structure was achieved after thermal annealing since the LC phase of the F8BT sample was present above approximately 125 °C. After removing the UV epoxy at room temperature (Fig. 1f), the hole-blocking layer (2,2',2"-(1,3,5-benzine triyl)-tris(1-phenyl-1-H-benzimidazole), TPBi), electron injection layer (LiF), and cathode (Al) were thermally deposited for EL measurement (Fig. 1g and see Fig. S2 in Supplementary Information). The PL measurements were obtained from samples without electrodes and other supporting layers. All processes were carried out in a glove box filled with N_2_ gas to avoid exposure to humidity and oxygen. The effect of the second rubbing process on the upper F8BT surface and the role of the UV epoxy as the protecting film were investigated with atomic force microscopy (AFM) and scanning electron microscopy (SEM) (see Figs. S3 and S4 in Supplementary Information). From the AFM results and the corresponding Fourier transformation as shown in Fig. 1g, we clearly observed that the anisotropic surface morphology of the F8BT layer is maintained after removing NOA65.

**Fig. 2:**
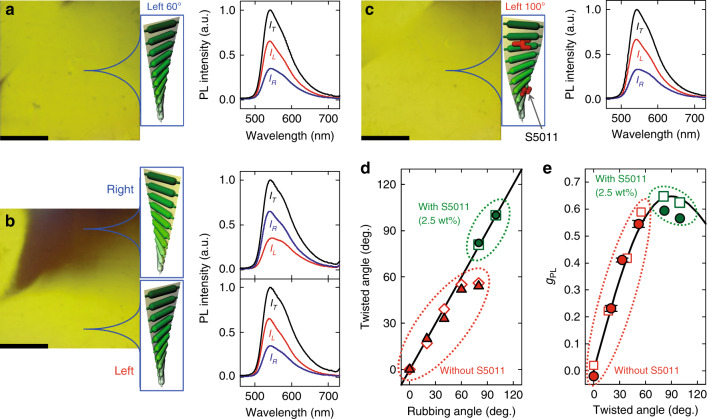
The alignment textures and PL spectra. **a**–**c** Alignment textures, illustrations of the corresponding twisted structures and PL spectra for various rubbing angles *R*_*θ*_ (**a** *R*_*θ*_ = 60° and **b** *R*_*θ*_ = 90° without chiral dopant, and **c** *R*_*θ*_ = 100° with 2.5 wt% of chiral dopant). The PL spectra measured without a circular polarizer, and with LH and RH circular polarizers are presented by black (*I*_T_), red (*I*_L_), and blue (*I*_R_) solid lines, respectively. The dark and bright areas in (**b**) indicate that RH and LH twist structure, respectively. For the comparison, the spectrum intensities were normalized with respect to the peak intensity without a circular polarizer. **d** Measured twisted angle of the F8BT layer as a function of the rubbing angle. Open diamonds and filled triangles are measured from the F8BT layers without chiral dopant before and after removing NOA65, respectively. Open squares and filled circles are measured from the F8BT layer blended with 2.5 wt% of S5011 before and after removing NOA65, respectively. **e** |*g*_PL_| as a function of the twisted angle. The symbols represent experimentally measured values from the CPPL (open squares and filled circles are the values before and after removing NOA65, respectively) and the solid line represents calculated value using the Mueller matrix method. Here, red (red open squares and red filled circles) and green symbols (green open squares and green filled circles) represent the *g*_PL_ values of the pure F8BT without the chiral dopant and those of the F8BT with the 2.5 wt% chiral dopant. Here, all scale bars represent 0.5 mm.

The alignment textures and the corresponding PL spectra for various rubbing angles (*R*_*θ*_), defined by the second F8BT rubbing direction with respect to the first PI rubbing direction, are shown in Fig. 2. Since the CP light is emitted when LP light passes through the twisted sample, the handedness of the emitted CP light directly corresponds to the twisted sense of the F8BT sample under our cell structures (see Fig. S5 in Supplementary Information for the experimental setup for the observation). As shown in Fig. 2a, the F8BT sample with *R*_*θ*_ = 60° exhibits a uniform alignment texture, which means that the twist in the sample occurs uniformly in one direction (here, leftward). As a result, the measured PL spectra show that the LHCP light has a higher intensity than the RHCP light in the entire range of measured wavelengths. On the other hand, we clearly observed two domains in the F8BT sample with *R*_*θ*_ *=* 90°, as shown in Fig. 2b. The dark and bright areas indicate the RHCP light in the rightward twisted structure and the LHCP light in the leftward twisted structure, respectively. In addition, the PL spectra show the intensity inversion in the two domains. Since the tendencies of the rightward and leftward twists are both *R*_*θ*_ = 90° in the sample, two domains with different twisted senses simultaneously occur. To give priority to the twisted sense and additional force, a small amount of chiral dopant can be introduced to the F8BT layer, which is well known in twisted nematic LC displays^8,26^. Figure 2c shows the alignment textures and the corresponding PL spectra of a sample with *R*_*θ*_ = 100°, in which F8BT is mixed with 2.5% of the chiral dopant S5011. Since a uniform bright region is formed in the entire sample region, the leftward twisted structure occurs, and thus, the PL spectra show that the LHCP light is stronger than the RHCP light at all measured wavelengths.

The twisted angles (*T*_*θ*_) of the F8BT sample as a function of the rubbing angle *R*_*θ*_ are shown in Fig. 2d. The twisted angles before (open symbols) and after (filled symbols) removing the NOA65 are almost equivalent to each other for all rubbing angles. When the *R*_*θ*_ < 60°, the *T*_*θ*_ is linearly proportional to the *R*_*θ*_, but the *T*_*θ*_ is saturated at *θ*_s_ = 56° when 60° < *R*_*θ*_ < 90° (open diamonds and filled triangles). Since the twisted elastic constant (*K*_2_ ≈ 10^−10^ ~ 10^−11^ N) of the polymer is approximately 10−100 times larger than that (*K*_2_ ≈ 10^−12^ N) of the conventional LC molecules used in the LC displays^29,30^, the *T*_*θ*_ is limited by the competition between the elastic energy and the surface anchoring energy (*W*_*θ*_) when the *R*_*θ*_ is larger than a saturated angle (*θ*_s_). The surface anchoring energy is roughly estimated as *W*_*θ*_ = *K*_2_*θ*_s_^2^/2*d*, where *d* is the thickness of the EML^31^. With *d* = 200 nm, *θ*_s_ = 56°, and *K*_2_ = 10^−10^ ~ 10^−11^ N, the anchoring energy is evaluated as 10^−4^ ~ 10^−5^ J/m^2^, which is comparable to that of the conventional alignment layers used in LC displays. Due to the high elastic constant of F8BT, additional force must be introduced to achieve *T*_*θ*_ > *θ*_s_ by overcoming the twisted elastic energy. The leftward twist and additional force were generated by adding a small amount of the chiral dopant S5011 with a high helical twisting power (HTP) of approximately 100 μm^−1^ for conventional LC molecules^32^. As shown in Fig. 2d, the twisted angle (open squares and filled circles) is linearly proportional to the rubbing angle even above 60° by adding 2.5 wt% of S5011 to F8BT. Note that the 2.5 wt% dopant intrinsically produces a twist of the F8BT layer of approximately 40° (see Fig. S6 in Supplementary Information). Consequently, we concluded that the twisted stacking of F8BT is introduced by different boundary conditions on the upper and lower surfaces without any chirality.

The measured and calculated *g* values for the PL (*g*_PL_) with various twisted angles are shown in Fig. 2e. Here, open squares and filled circles represent the *g*_PL_ values before and after removing the NOA65 film, respectively. Red symbols (red open squares and red filled circles) and green symbols (green open squares and green filled circles) represent the *g*_PL_ values of the pure F8BT without the chiral dopant and those of F8BT with the 2.5 wt% chiral dopant, respectively. For given sample properties such as the total twisted angle and film thickness, the *g*_PL_ factors for several samples were measured at similar values in the same polymer batch, as shown in Fig. 2e. For cases both with and without the chiral dopant, the *g*_PL_ values exhibit similar behavior. In a circularly dichroic medium, the dissymmetric *g* value can also be measured. We investigated circular dichroism of the twisted F8BT layer and found that the resultant circular dichroism was very small to generate the large *g* value measured in this work (see Fig. S7 in Supplementary Information). Consequently, we reconfirmed that a large *g* value originated from the twisted stacking of the F8BT sample without any chirality or circular dichroism. The *g*_PL_ value increased with increasing twisted angle up to 90° and gradually decreased with further increases in the twisted angle. The *g*_PL_ value was directly calculated from Stokes parameters using the Mueller matrix for the twisted birefringent material (see Mueller matrix analysis in Supplementary Information and refs. ^11^ and ^33^ for further details). For calculation of the *g*_PL_ value, we assume that the F8BT film is uniformly twisted and divided into *N* sublayers. In the PL process, LP light is emitted at each sublayer and propagated in the twisted medium, which is described by the Mueller matrix. Here, the LP light is partially polarized since the F8BT film is not aligned perfectly. As a result, the measured *g*_PL_ values were limited by the degree of polarization of the light (*P*_PL_), and thus, the calculated *g*_PL_ values were expressed by *g*_PL_ = *P*_PL_ × *g*_ideal_, where *g*_ideal_ is a *g* value calculated from the above Mueller matrix analysis for completely polarized light. The solid line in Fig. 2e represents the *g*_PL_ with *P*_PL_ = 0.72, which describes the experimental data well. It should be noted that the *P*_PL_ was determined by a dissymmetric ratio of intensity parallel and perpendicular to the polarizer in the sample with *T*_*θ*_ = 0° (see Fig. S8 in Supplementary Information). Additionally, the *g*_PL_ value was maintained in a wide temperature range from 10 to 80 °C without degradation (see Fig. S9 in Supplementary Information). It should be noted that in many commercial applications, OLEDs need to remain stable when stored at a temperature of 85 °C for extended periods of time. Therefore, it is very important that the twisted structure and the *g* value are maintained at high temperatures^21^.

**Fig. 3:**
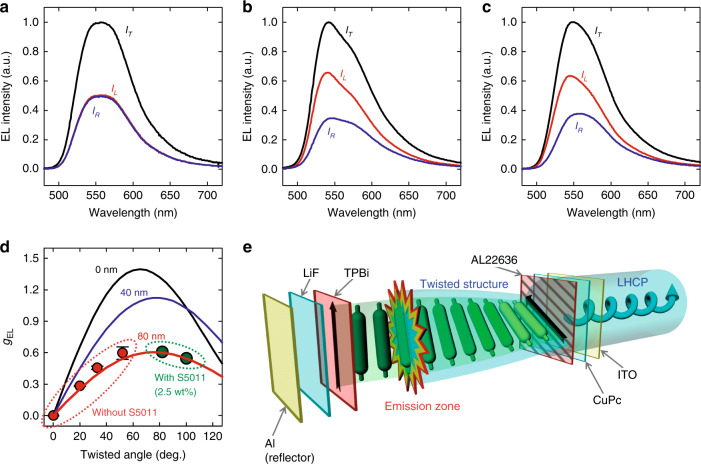
EL spectra and g-factors. **a**–**c** EL spectra for various twisted angles *T*_*θ*_ (**a** *T*_*θ*_ = 0° and **b** *T*_*θ*_ = 52° without chiral dopant, and **c** *T*_*θ*_ = 100° with 2.5 wt% of chiral dopant). The EL spectra measured without a circular polarizer, and with LH and RH circular polarizers are presented by black (*I*_T_), red (*I*_L_), and blue (*I*_R_) solid lines, respectively. **d** |*g*_EL_| as a function of the twisted angle. The filled circles represent experimentally measured values from the CPEL (red and green symbols represent the *g*_EL_ values of the pure F8BT without the chiral dopant and those of the F8BT with the 2.5 wt% chiral dopant). Using the Mueller matrix analysis, |*g*_EL_| calculated as a function of the twisted angle at different recombination zone of 0 (black solid line), 40 (blue solid line), and 80 nm (red solid line) from the TPBi. **e** Schematic diagram of the CPEL mechanism.

To confirm the applicability of the second rubbing process to the fabrication of the EL devices, CPEL devices were fabricated, as shown in Fig. 1. The CPEL spectra and the resultant *g*_EL_ values for the EL devices with different twisted angles are shown in Fig. 3. As expected, pure F8BT with *T*_*θ*_ = 0° did not emit CP light but emitted LP light, as shown in Fig. 3a. From an intensity ratio of the LP light, the degree of polarization (*P*_EL_) in the EL process was determined to be 0.81 at a wavelength of 546 nm (see Fig. S8 in Supplementary Information). On the other hand, in the pure F8BT with *T*_*θ*_ = 52° (Fig. 3b) and F8BT with *T*_*θ*_ = 100° by adding 2.5 wt% of chiral dopant (Fig. 3c), dissymmetric spectra were obviously observed. Consequently, the *g*_EL_ value gradually increased with increasing twisted angle up to 60° and decreased with further increasing twisted angle, as shown in Fig. 3d. For given sample conditions, the *g*_EL_ values for several samples were measured to be similar to the *g*_PL_ values. The efficiency of the CPEL device in which the second rubbing process was applied was slightly reduced from 1.0 to 0.9 cd/A (see Fig. S10 in Supplementary Information). Such reduction presumably originates from the presence of a rough interface by the rubbing process and contaminants by the peeling of NOA65 (see Figs. S3 and S4 in Supplementary Information). It should be noted that the interface roughness can be reduced by using a noncontact alignment method, such as photoalignment^34,35^.

In the case of the EL process, the *g*_EL_ value was also directly calculated with Mueller matrix analysis for the twisted birefringent sublayers used in the PL process except that the LP light emitted only at specific sublayers, indicating an electron-hole recombination zone (emission zone). Figure 3e shows a schematic diagram of the emission of LHCP light in the EL process. The LP light emitted at the emission zone propagates towards the anode and cathode with the same probability and passes through the twisted birefringent sublayers. In particular, the LP light emitted towards the cathode is reflected from the cathode (reflector), and thus, the reflected light passes through all the twisted birefringent sublayers (see Mueller matrix analysis in Supplementary Information and refs. ^11^ and ^33^ for further details). As a result, the *g*_EL_ values were calculated by averaging the *g*_EL_ values for both directions. In the EL process, it is important to consider an emission zone (electron-hole recombination zone); thus, it is necessary to calculate the *g*_EL_ value depending on the emission zone^11^. For simplifying the calculation, we did not consider the attenuation of intensity during light propagation^36^. In Fig. 3d, solid lines represent the calculated *g*_EL_ values when the emission zone is located at different distances (*z*) from the TPBi. Notably, the TPBi layer is isotropic, and thus, there is no change in the polarization state. It can be clearly seen that the calculated values with *z* = 80 nm agree well with the experimental results. For the calculation, we used Δ*n* *=* 0.623 at *λ* = 546 nm, *P*_EL_ = 0.81, and *d* *=* 200 nm. The *g*_EL_ was measured to be approximately 0.6 in the 60°-twisted sample without a chiral dopant. Consequently, the external quantum efficiency of the OLED under the circular polarizer is enhanced by 30% compared to that of the conventional OLED with random polarization (see Fig. S1 in Supplementary Information). Since the location of the recombination zone can be controlled by the thickness of the hole-blocking layer (TPBi)^33^, the *g*_EL_ value can be enhanced by optimizing the device structure.

## Discussion

**Fig. 4:**
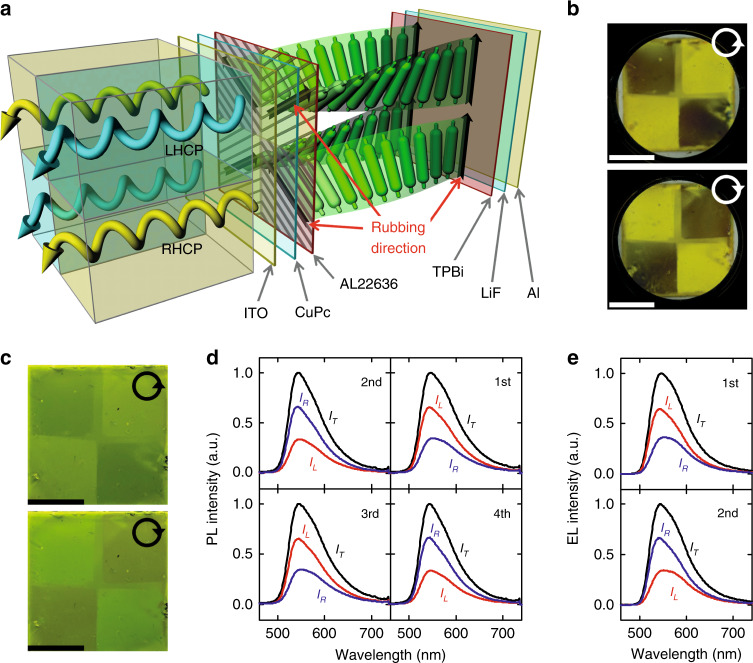
The orthogonal CPPL and CPEL from the single EML. **a** Schematic diagram of the simultaneous emission with orthogonal handedness in circular polarization from single emitting layer. The multidirectionally rubbed AL22636 surface and the unidirectionally rubbed F8BT surface produce the reverse twisted structures in the single EML. **b** Microscopic textures and **c** PL textures under LH (top image) and RH (bottom image) circular polarizers. **d** The corresponding CPPL spectra in each quadrant in (**c**). **e** The CPEL spectra of the EL sample for the first (top spectra) and second (bottom spectra) quadrants. All spectra measured without a circular polarizer, and with LH and RH circular polarizers are presented by black (*I*_T_), red (*I*_L_), and blue (*I*_R_) solid lines, respectively. Here, all scale bars represent 1 cm.

Now, we demonstrated the simultaneous emission of LHCP light and RHCP light from a single luminophore in a single device using the second rubbing process. It should be noted that a chiral moiety (including the dopant) was required to generate the CP light, and thus, only either LHCP light or RHCP light was obtained in a single device. To fabricate a multidomain CPEL device, we used a multirubbing process on the polyimide (the first rubbing process) through a shadow mask to implement multidomains. For the first rubbing process (lower surface of the EML), the polyimide film was rubbed at −60° in the first and third quadrants of the sample and at +60° in the second and fourth quadrants. After spin-coating F8BT on the multirubbed polyimide, the whole surface of the F8BT layer was rubbed at 0° for the second rubbing process (upper surface of the EML). Finally, the simultaneous emission of LHCP light and RHCP light from a single luminophore in a single device was achieved, as shown in Fig. 4a. The alignment textures and corresponding PL textures under the LH and RH circular polarizers are shown in Fig. 4b, c, respectively. It is clear that the F8BT domains in the first and third quadrants are brighter than those in the second and fourth quadrants, and thus, the first and third quadrants form a leftward twisted structure, but the second and fourth quadrants form a rightward twisted structure. From the CPPL spectra shown in Fig. 4d, we obtain *g*_PL_ = 0.61 and 0.60 for the first and third quadrants, respectively, but *g*_PL_ = −0.63 and −0.64 for the second and fourth quadrants. Note that the minus sign means that RHCP light is stronger than LHCP light and vice versa for the plus sign. Similarly, from the CPEL spectra shown in Fig. 4e, we obtain *g* values with different signs in two EL devices corresponding to the first (*g*_EL_ = 0.57) and second (*g*_EL_ = −0.64) quadrant in the EL devices.

In summary, the different boundary conditions on the two surfaces of the mesogenic EML produce a twisted configuration with the achiral conjugate polymer, with no chiral components added, due to its elasticity. The resultant macroscopic twisted configuration generates CP light with a high dissymmetry factor in both the PL and EL processes. Moreover, by controlling the different alignment directions on the surfaces of the EML, patterned CP light with various polarization states can be achieved through the fabricating process proposed herein. This experimental demonstration highlights the feasibility of the light source with multipolarization, including orthogonal CP states, thereby paving the way towards novel applications in biosensors as well as optical devices such as OLEDs.

## Materials and methods

### Fabrication of OLEDs

As an emitting layer for CP emission, the LC conjugate polymer of F8BT (from Solaris Chem Inc., Quebec, Canada) was dissolved in toluene (28.7 mg/ml) for spin coating. Copper phthalocyanine (CuPc from LUMTEC, New Taipei City, Taiwan, China) with a thickness of 2 nm was deposited by high-vacuum (6 × 10^−6^ torr) thermal evaporation for hole injection on prepatterned indium tin oxide (ITO). For uniform alignment of the F8BT, polyimide (PI) (AL22636 from JSR, Cheong-Ju, Korea) was spin-coated for hole transport on the CuPc and rubbed by a rubbing machine (RMS-50-M from Nam Il Optical Instruments Co.) with a 6.5-cm-diameter roller covered with cotton cloth, whose pile length and number of piles were 1.8 mm and 3750 piles/inch^2^, respectively. The rotational speed of the roller and the translational speed of the substrate stage were fixed at 500 rpm and 6 mm/s, respectively^28,33^. The dissolved F8BT with a thickness of 200 nm was spin-coated at 3000 rpm for 20 s followed by 1000 rpm for 10 s on the rubbed PI. The F8BT layer was unidirectionally rubbed with directions different from the PI rubbing direction. Next, an ultra-violet (UV)-curable resin (NOA65 from Norland Product Inc.) was covered on the F8BT sample and cured by UV exposure. The NOA65 layer was peeled off from the F8BT sample after annealing at 150 °C for 10 min to induce a twisted structure due to the elastic properties of the LC conjugate polymer. Finally, TPBi (20 nm), LiF (1 nm), and Al (70 nm) were sequentially deposited by high-vacuum (6 × 10^−6^ torr) thermal evaporation as a hole-blocking layer to confine excitons within the EML, electron injection layer, and cathode, respectively. All EL samples were encapsulated by glass and NOA65 to avoid exposure to humidity and oxygen. The schematic diagrams of the OLED structure and energy levels and the chemical structures of the materials used are shown in Fig. S2.

### Characterization

The CP emission spectra were collected under a circular polarizer consisting of a linear polarizer and a quarter-wave plate at 546 nm using a spectroradiometer (SR-UL 1R from TOPCON). The current density (*J*)−voltage (*V*)−luminance (*L*) characteristics of the OLEDs were evaluated using a spectroradiometer, a programmable power supply (PPE-3323 from GW Instek), and a multimeter (Keithley 2000 from Keithley Instruments Inc.). The alignment texture and the surface morphology were observed by a polarized microscope (E600W POL from Nikon) with a frame-grabbing system (SDC-450 from Samsung), AFM (XE-100 from Park System), and SEM (Nova Nano SEM 200 from FEI). The twisted angle was determined by direct measurement of the Stokes parameters of the transmitted light as described in refs. ^20^ and ^28^.

## Supplementary information

Supplementary Information for Simultaneous emission of orthogonal handedness in circular polarization from a single luminophore
