# Supplementary material for: Simultaneous emission of orthogonal handedness in circular polarization from a single luminophore
Source: Light Sci Appl. 2019 Dec 12;8:120. doi: 10.1038/s41377-019-0232-0 (PMC6908657; doi:10.1038/s41377-019-0232-0)
Supplement: Supplementary file 1 — Supplementary Information for Simultaneous emission of orthogonal handedness in circular polarization from a single luminophore [file 41377_2019_232_MOESM1_ESM.docx]

Supplementary Information for

Simultaneous emission of orthogonal handedness in circular polarization
from a single luminophore

Kyungmin Baek^1^, Dong-Myung Lee^1^, Yu-Jin Lee^1^, Hyunchul Choi^2^, Jeongdae Seo^2^, Inbyeong Kang^2^, Chang-Jae Yu^1*^, and Jae-Hoon Kim^1#^

^1^Department of Electronic Engineering, Hanyang University, 222 Wangsimni-ro, Seongdong-gu, Seoul 04763, Republic of Korea

^2^LG Display Co., Ltd., LG Science Park, 30 Magokjungang 10-ro, Gangseo-gu, Seoul 07796, Republic of Korea

E-mail *[cjyu@hanyang.ac.kr](mailto:cjyu@hanyang.ac.kr) ; # [jhoon@hanyang.ac.kr](mailto:jhoon@hanyang.ac.kr)

**1. Efficiency of OLED Depending on the Degree of Circular Polarization**

In a conventional OLED, since a circular polarizer in front of the OLED panel is inevitably required to prevent reflection of ambient light from a metal electrode, only half of the light extracted from the OLED panel reaches to eyes. That is, the maximum efficiency of the emitted light is about 50 % even other losses such as internal reflection are not taken into account. As a result, direct emission of CP light from OLED with the same handedness as the circular polarizer in front of the OLED panel can increase the efficiency of the emitted light without any light extracting structure. Therefore, the higher dissymmetry *g* factor gives rise to the higher performance in the OLED.


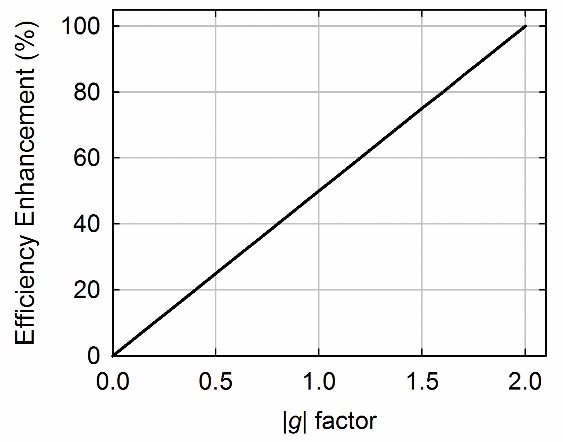


**Figure S1**. Efficiency enhancement of the OLED as a function of the dissymmetry *g* factor.

The dissymmetry *g* factor is defined by

$$g= \frac{2\left( I_{L}-I_{R} \right)}{I_{L}+I_{R}}$$

Here, *I_L_* and *I_R_* denote the intensities of LHCP and RHCP light, respectively. The |*g*| factor is bounded by 2 and its sign depicts the handedness of the CP light. When total intensity is normalized, sum of *I_L_* and *I_R_* is unity. Therefore, the LHCP intensity *I_L_* is expressed by the *g* value:

$$I_{L}= \frac{2+g}{4}$$

Since the LHCP component of the conventional light with random polarization is 0.5, the enhancement of the efficiency in the OLED under LHCP for preventing reflection of ambient light from a metal electrode is described by a ratio of the enhancement to the LHCP component of the random polarized light:

$$\eta= \frac{g}{2}\times100 (\%)$$

With increasing the |*g*| value, enhancement of the efficiency is linearly increased up to 100 %, which means twice enhancement compared to the conventional OLED.

**2. Device Structure and Materials**

The OLED structure and energy levels of the materials, including molecular structure, used here are shown in **Figure S2**. The polyimide of AL22636 and the F8BT were spin-coated, but the others were deposited by thermal evaporation.


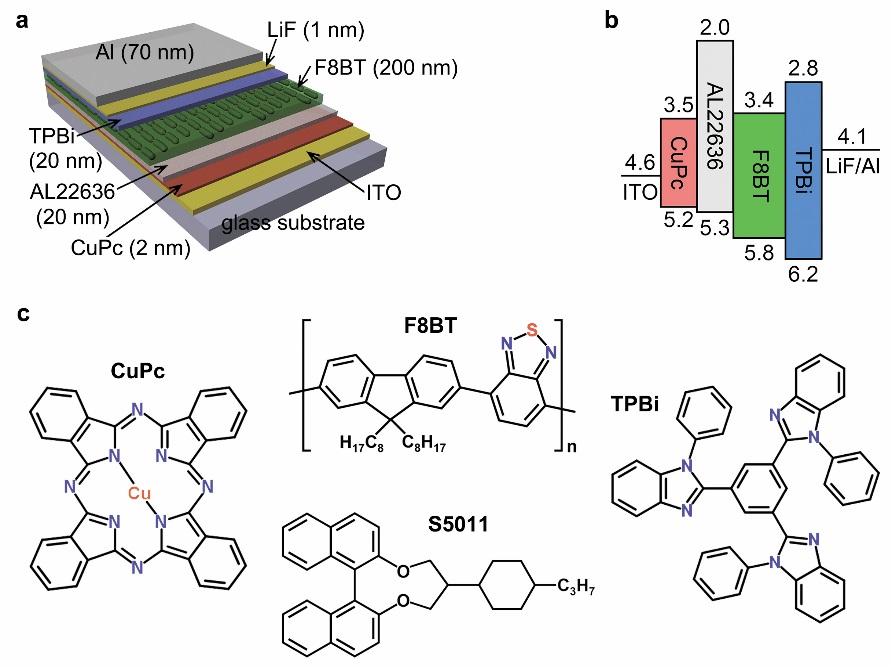


**Figure S2**. (a) The schematic diagram and (b) the corresponding energy levels of the OLED. (c) The molecular structures of the materials used here. CuPc: Copper phthalocyanine; F8BT: poly(9,9-di-n-octylfluorenyl-2,7-diyl)-alt-(benzo[2,1,3]thiadiazol-4,8-diyl)]; TPBi: 2,2',2"-(1,3,5-benzine triyl)-tris(1-phenyl-1-H-benzimidazole).

**3. Surface Anchoring Effect by UV Epoxy**

We investigated the surface morphology of the rubbed upper surface with an atomic force microscope (AFM) to confirm the 2^nd^ rubbing effect and the role of UV epoxy. Prior to the 2^nd^ rubbing, the surface of the coated F8BT is smooth and isotropic (**Figure S3**a), but the polymer clusters extending in the direction of rubbing are clearly visible after the 2^nd^ rubbing in **Figure S3b**. The anisotropy of the surface morphology, shown in the corresponding Fourier transformed spectra, is believed to generate surface anchoring energy that aligns the polymer chains in a direction different from the 1^st^ rubbing. However, since the F8BT does not exhibit a LC phase at room temperature, the sample was annealed at 150 °C above the glass transition temperature (*T_g_* = 125 °C) for 10 min and quenched to room temperature in order to keep their alignment. **Figure S3c** clearly shows that anisotropic morphology (groove structure) induced by the 2^nd^ rubbing disappears after thermal annealing, and a smooth surface structure was formed as same as that of before rubbing. The anchoring force by rubbing is diminished during the thermal annealing. In order to keep the anisotropic surface by the 2^nd^ rubbing, we coated UV epoxy (NOA65 from Norland Product Inc.) and peeled it off after thermal annealing. We clearly observed that the anisotropic surface morphology after removing NOA65 is maintained even after thermal annealing shown in **Figure S3d**. We believe that the anisotropic state of the surface migrated to NOA65, and this anisotropic state of NOA would orient the polymer chain during the thermal annealing.


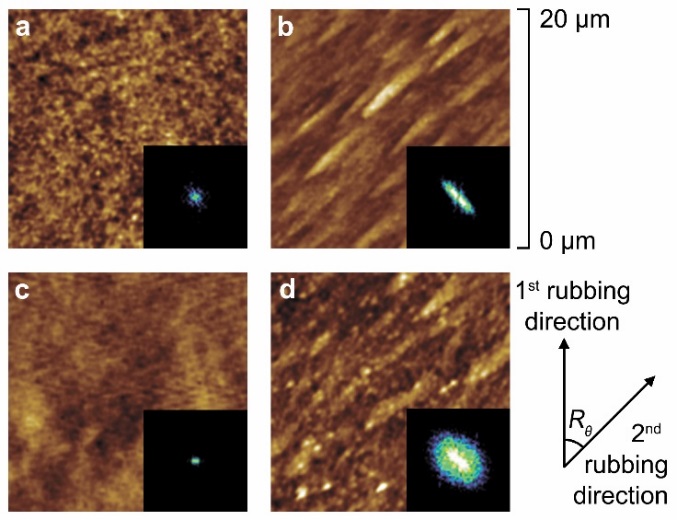


**Figure S3**. AFM images of the F8BT surfaces with (a) prior to the 2^nd^ rubbing and (b) after the 2nd rubbing. The images of thermally annealed F8BT surfaces (c) without and (d) with NOA65 layer. The arrows are indicating the direction of the 1^st^ and the 2^nd^ rubbing. The insets exhibit Fourier transformed spectra images.

We also investigated the surface morphology of the rubbed upper surface with a scanning electron microscope (SEM) to confirm the 2^nd^ rubbing effect. The rubbed F8BT surface for SEM image was prepared by the same procedure as: coating the F8BT → rubbing the upper surface of the F8BT → coating the NOA65 onto the rubbed F8BT layer → thermal annealing the sample and cooling it down → peel it off → observing the SEM image. **Figure S4** clearly shows the groove structure on the upper F8BT surface. However, some contaminants, which might be originated from the NOA65 during peeling it off, were observed. Such contaminants might give rise to subtle degradation of the EL performance (see **Figure S10**).


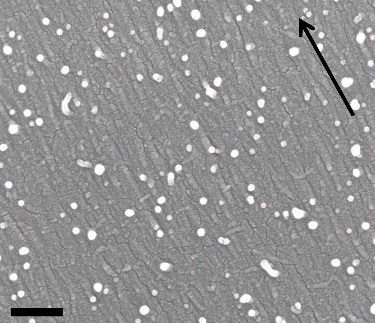


**Figure S4**. SEM image of the rubbed F8BT surfaces after peeling off NOA65. Here, scale bar represents 200 nm and arrow is indicating the rubbing direction.

**4. Optical Setup for Observation of Alignment Textures**

Polarized optical microscope (E600W POL from Nikon) with frame-grabbing system (SDC-450 from Samsung) is used to observe the alignment textures of the F8BT layer. The textures under circular polarizer were obtained by inserting a quarter-wave-plate (QWP) between the sample and the analyzer as shown in **Figure S5**.


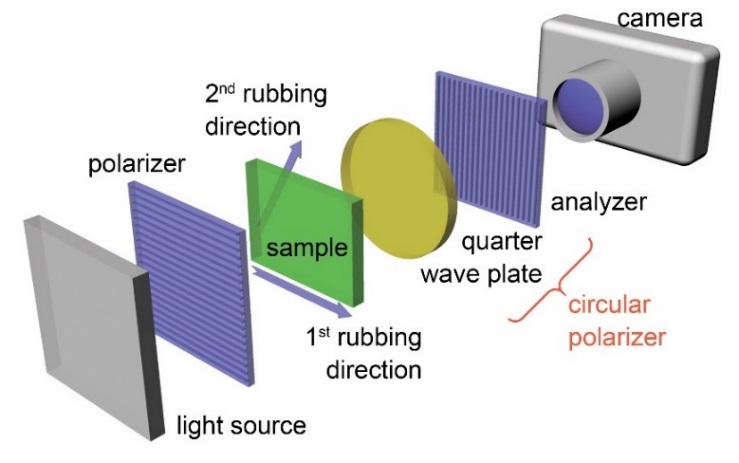


**Figure S5**. Optical set-up for observing the alignment texture. The linearly polarized white light parallel to the 1^st^ rubbing direction passes through the sample and observed on the camera through a circular polarizer.

**5. Twisting Power of the Chiral Dopant S5011**

The LC molecules are continuously twisted by adding the chiral dopant. The helical pitch (*p*) of the twisted LC phase is inversely proportional to the blended concentration (*c*) of the chiral dopant as follows ^[1]^:

$$p=\frac{1}{HTP\times c}$$

Here, *HTP* represents the helical twisting power of the chiral dopant for the LC molecules. For a given thickness (*d*) of the twisted EML layer, the twisted angle (*θ_T_*) is expressed as

$$\theta_{T}=2\pi d\cdot HTP\times c$$

From the least-square-fit of the measured twisted angles to the above equation, the HTP was calculated to be 20.4 μm^-1^ as shown in **Figure S6**. Here, the twisted angle of the F8BT was evaluated from the Stokes parameters based on the matrix analysis for the twisted birefringent media ^[2]^.


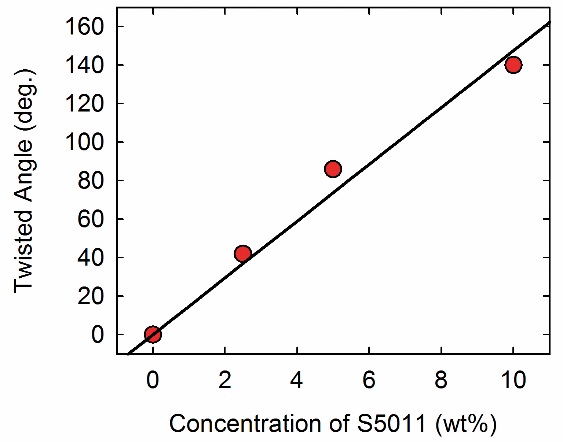


**Figure S6**. The twisted angle as a function of the blending concentration of the chiral dopant S5011. The symbols and the solid line represent the experimental results and the least-square fit to the above equation.

**6. Circular Dichroism of the F8BT layer**

We investigate the circular dichroism of the twisted F8BT layer, to confirm that a large *g* factor is originated from the twisted geometry rather than the circular dichroism of the F8BT. The sample for the circular dichroism was prepared with peeling off the NOA65 after thermal annealing of the rubbed F8BT layer as mentioned in **Section 3**. Surface Anchoring Effect by UV Epoxy. As shown in **Figure S7**, the absorption spectra under right-handed circular polarizer (RHCP) and left-handed circular polarizer (LHCP) almost coincide with each other. The resultant circular dichroism was very small to generate the large *g* value measured in this work. Consequently, we reconfirmed that large *g* value was originated from the twisted stacking of the F8BT without any chirality nor circular dichroism.


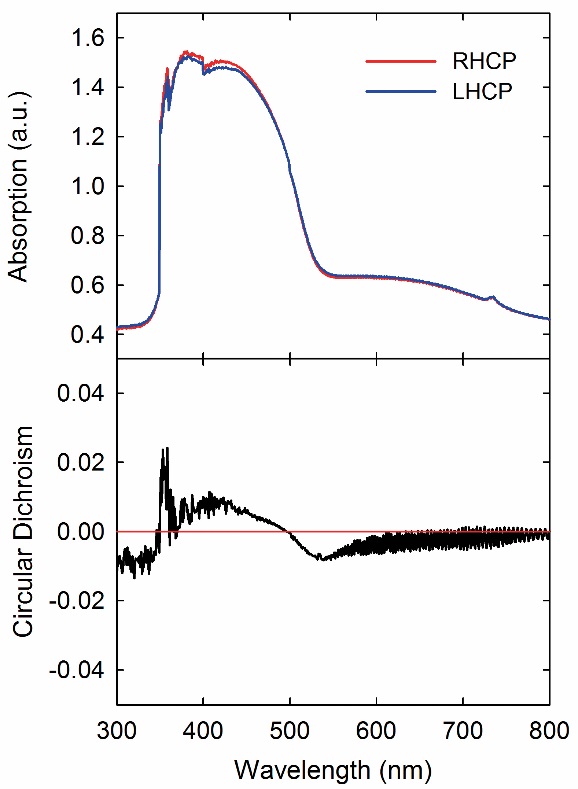


**Figure S7**. Absorption spectra under RHCP and LHCP, and the resultant circular dichroism of the twisted F8BT layer.

**7. The Degree of Polarization for the PL and EL Processes**

The degrees of polarization in the PL (*P_PL_*) and EL (*P_EL_*) processes are determined by dissymmetric ratios of intensity parallel (*I*_0_) and perpendicular (*I*_90_) to the polarizer in the sample with *T_θ_* = 0°, which is achieved in the sample without the 2^nd^ rubbing process as follows:

$$P_{PL,EL}=\frac{I_{0}-I_{90}}{I_{0}+I_{90}}$$

From both PL and EL spectra shown **in Figure S8**, the degrees of polarization in the PL and EL processes are evaluated to be 0.72 and 0.81 at a wavelength of 546 nm, respectively. The *g* value is bounded by these degrees of polarization.


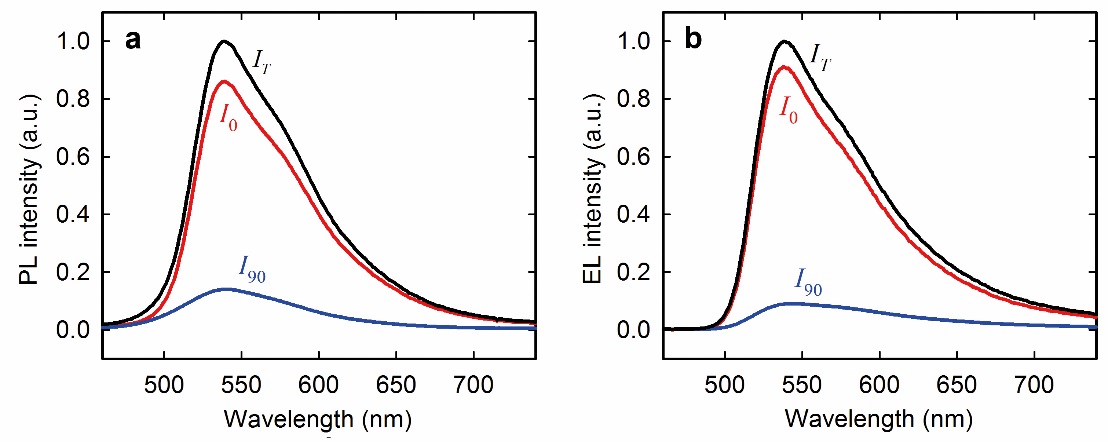


**Figure S8**. (a) LPPL and (b) LPEL spectra for the pure F8BT film (*d* = 200 nm) without the 2^nd^ rubbing process (*T_θ_* = 0°). The PL and EL spectra measured without a linear polarizer, and with 0° and 90° polarizers are presented by black (*I*_T_), red (*I*_0_), and blue (*I*_90_) solid lines, respectively. For the comparison, the intensities were normalized with respect to the peak intensity without a linear polarizer.

**8. Mueller Matrix Analysis of the *g_ideal_* Factor in the PL and EL processes**

**8.1 The *g_ideal_* Factor in the PL process**

The polarization state of light propagating the birefringent medium is easily expressed by the Stokes parameters **S**, which is calculated by Mueller matrix representing the birefringent medium. For calculation of the *g* factor, we assume that the F8BT layer is uniformly twisted in the film and divided to *N* sublayers ^[3]^. Now, the twisted angle of the *i*-th sublayer depicts *θ_i_* = (*Φz_i_*)/*d*, where *Φ*, *z_i_*, and *d* are total twisted angle, distance from the hole transport layer, and the film thickness, respectively. The Mueller matrix for the *i*-th sublayer with a phase retardation Γ is

$$\mathbf{M}\left( \Gamma,\theta_{i} \right)=\left( \begin{matrix} 1 & 0 & 0 & 0 \\ 0 & \cos\left( 2\theta_{i} \right) & -\sin\left( 2\theta_{i} \right) & 0 \\ 0 & \sin\left( 2\theta_{i} \right) & \cos\left( 2\theta_{i} \right) & 0 \\ 0 & 0 & 0 & 1 \end{matrix} \right)\left( \begin{matrix} 1 & 0 & 0 & 0 \\ 0 & 1 & 0 & 0 \\ 0 & 0 & \cos\left( \Gamma\right) & -\sin\left( \Gamma\right) \\ 0 & 0 & \sin\left( \Gamma\right) & \cos\left( \Gamma\right) \end{matrix} \right)\left( \begin{matrix} 1 & 0 & 0 & 0 \\ 0 & \cos\left( 2\theta_{i} \right) & \sin\left( 2\theta_{i} \right) & 0 \\ 0 & -sin \left( 2\theta_{i} \right) & \cos\left( 2\theta_{i} \right) & 0 \\ 0 & 0 & 0 & 1 \end{matrix} \right)=\left( \begin{matrix} 1 & 0 & 0 & 0 \\ 0 & \cos^{2} \left( 2\theta_{i} \right)+\sin^{2} \left( 2\theta_{i} \right)\cos\left( \Gamma\right) & \cos\left( 2\theta_{i} \right)\sin\left( 2\theta_{i} \right)\left\{ 1-\cos\left( \Gamma\right) \right\} & \sin\left( 2\theta_{i} \right)\sin\left( \Gamma\right) \\ 0 & \cos\left( 2\theta_{i} \right)\sin\left( 2\theta_{i} \right)\left\{ 1-\cos\left( \Gamma\right) \right\} & \sin^{2} \left( 2\theta_{i} \right)+\cos^{2} \left( 2\theta_{i} \right)\cos\left( \Gamma\right) & -\cos\left( 2\theta_{i} \right)\sin\left( \Gamma\right) \\ 0 & -\sin\left( 2\theta_{i} \right)\sin\left( \Gamma\right) & \cos\left( 2\theta_{i} \right)\sin\left( \Gamma\right) & \cos\left( \Gamma\right) \end{matrix} \right)$$

In the PL process, the UV light was absorbed and the visible light was emitted at the *j*-th sublayer. The emitted light experiences the twisted birefringent medium from the *j*-th sublayer to the *N*-th sublayer.

$$\mathbf{S}_{jO}=\left( \prod_{i=j}^{N} \mathbf{M}\left( \Gamma,\theta_{i} \right) \right)\mathbf{S}_{j}$$

Here, **S***_j_* and **S***_jO_* depict the Stokes vector of the emitted light at the *j*-th sublayer and that of the outgoing light, respectively. By the definition of the Stokes parameters, intensity *I_L_* of the left-handed circular polarization is (*S*_0_ – *S*_3_)/2 and the right-handed circular polarization is (*S*_0_ + *S*_3_)/2. Also, using the definition of the *g* factor, *g_ideal_* = –(2*S*_3_)/*S*_0_. We assume that the light was emitted with the same probability at all sublayers in the PL process. Therefore, the final *g_ideal_* factor was calculated by average over all **S***_jO_*. Using all measured parameters such as total twisted angle, total thickness, birefringence, and the degree of polarization, the *g* factor can be calculated as shown in **Figure 2e**.

**8.2 The *g_ideal_* Factor in the EL process**

For the *g* factor analysis in the EL process, it is critical to consider an emitting position (electron-hole recombination zone). At first, it is assumed that the linearly polarized light is emitted at *j*-th sublayer. That is, electron and hole are recombined at the uniformly aligned *j*-th sublayer and the linearly polarized light is emitted at the *j*-th sublayer. The emitted light is propagated toward anode and cathode with the same probability. The light emitted at the *j*-th sublayer toward anode experiences the twisted birefringent medium from the *j*-th sublayer to the *N*-th sublayer.

$$\mathbf{S}_{anode}=\left( \prod_{i=j}^{N} \mathbf{M}\left( \Gamma,\theta_{i} \right) \right)\mathbf{S}_{e}$$

Here, **S***_e_* and **S***_anode_* depict the Stokes vector of the emitted light at the *j*-th sublayer and that of the outgoing light to the anode, respectively. On the other hand, the light toward anode experiences reversely the twisted birefringent medium from the *j*-the sublayer to the 0-th sublayer (which is a transpose of the matrix multiplication from the 0-th sublayer to the *j*-th sublayer) and is reflected from the cathode. The reflected light experiences the whole twisted birefringent medium from the 0-th sublayer to the *N*-th sublayer.

$$\mathbf{S}_{cathode}=\left( \prod_{i=0}^{N} \mathbf{M}\left( \Gamma,\theta_{i} \right) \right)\left( \prod_{i=0}^{j} \mathbf{M}\left( \Gamma,\theta_{i} \right) \right)^{T}\mathbf{S}_{e}$$

Here, **S***_cathode_* depicts the Stokes vector of the outgoing light to the cathode. By the definition of the Stokes parameters, intensity *I_L_* of the left-handed circular polarization is (*S*_0_ – *S*_3_)/2 and the right-handed circular polarization is (*S*_0_ + *S*_3_)/2. Also, using the definition of the *g* factor, *g_ideal_* = –(2*S*_3_)/*S*_0_. We assume that the light emitted at a certain position within the emitting layer propagates toward anode or cathode with the same probability. Therefore, the final *g_ideal_* factor was averaged over both propagating lights toward the anode and cathode. Using all measured parameters such as the emitting position (*i*-th sublayer), total twisted angle, total thickness, birefringence, and the degree of polarization, the *g* factor can be calculated as shown in **Figure 3d**.

**9. Thermal Stability of the *g* value**

In most commercial applications of the OLEDs, the device temperature may rise up to 85 °C by joule heating when driving the OLED, and thus thermal stability of the device is very important. We measured the *g_PL_* of the pure F8BT with a rubbing angle of 60° as function of temperature. As shown in **Figure S9**, the *g_PL_* is maintained well up to 80 °C but gradually collapsed from 90 °C.


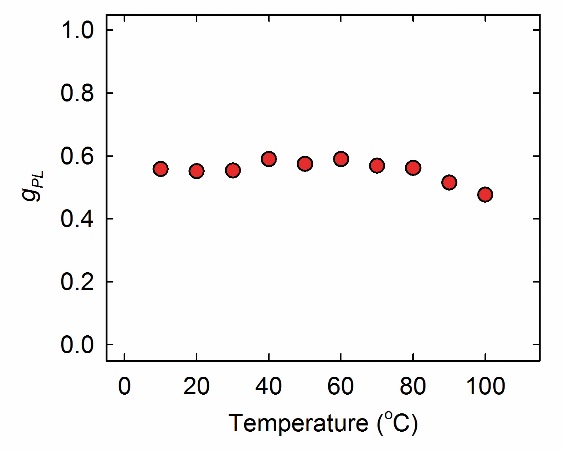


**Figure S9**. Thermal stability of the *g_PL_* with a rubbing angle of 60°. The twisted structure and the resultant *g_PL_* value were maintained well up to 80 °C.

**10. Efficiency of the CPEL Device with Twisted F8BT Layer**

The efficiency of the CPEL device was evaluated by using a spectroradiometer, a programmable power supply, and a multimeter. In the F8BT without applying the 2^nd^ rubbing process, turn-on voltage was 5 V and the maximum efficiency was 1.0 cd A^-1^ as shown in **Figure S10a**. In the F8BT with applying the 2^nd^ rubbing process (*R_θ_* = 60°), turn-on voltage was 8 V and the maximum efficiency was slightly reduced to be 0.9 cd A^-1^ (see **Figure S10b**). Such reduction is presumably originated from a rough interface by the rubbing process and contaminants by peeling off NOA65 as shown in **Figures S3 and S4**.


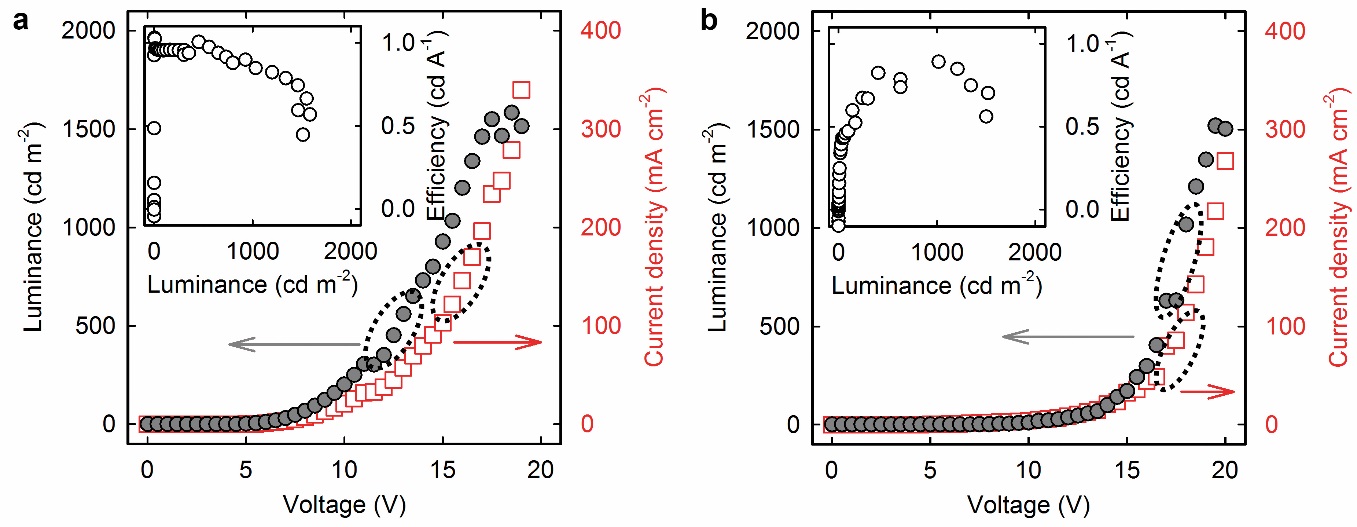


**Figure S10**. Current density (squares) and luminance (circles) versus voltage characteristics of the F8BT devices (a) without and (b) with applying the 2^nd^ rubbing process (*R_θ_* = 60°). Each inset figure represents efficiency versus luminance characteristics.

References

1. de Gennes, P. G. & Prost, J. R. The Physics of Liquid Crystals (Oxford Univ. Press, 1993).
2. Zhou, Y., He, Z. & Sato, S. A novel method for determining the cell thickness and twist angle of a twisted nematic cell by Stokes parameter measurement. *Jpn. J. Appl. Phys.* **36**, 2760-2764 (1997).
3. Kliger, D. S., Lewis, J. W. & Randall, C. E. Polarized light in optics and spectroscopy (Academic Press, 1990).
